# Non-invasive assessment of PD-L1 status and histology in non-small cell lung cancer using ^18^F-FDG PET/CT radiomics

**DOI:** 10.1186/s12967-026-08029-w

**Published:** 2026-03-18

**Authors:** Bartłomiej Tomasik, Marcin Jąkalski, Michał Bieńkowski, Jacek Teodorczyk, Bartosz Kamil Sobocki, Konrad Stawiski, Jacek Burzynski, Grzegorz Romanowicz, Rafał Dziadziuszko, Wojciech Cytawa, Jakub Mieczkowski

**Affiliations:** 1 Department of Oncology and Radiotherapy, Medical University of Gdańsk, Gdańsk, Poland; 2 Centre for Experimental CardioOncology, Medical University of Gdańsk, Gdańsk, Poland; 3 3P-Medicine Laboratory, Medical University of Gdańsk, Gdańsk, Poland; 4 Applied Genomics and Bioinformatics Centre, Faculty of Biology, University of Gdańsk, Gdańsk, Poland; 5 Department of Pathomorphology, Medical University of Gdańsk, Gdańsk, Poland; 6 Department of Nuclear Medicine, Medical University of Gdańsk, Gdańsk, Poland; 7 Department of Biostatistics and Translational Medicine, Medical University of Łódź, Łódź, Poland

**Keywords:** Non-small cell lung cancer, Radiomics, ^18^F-FDG PET/CT, PD-L1, Histology, Machine learning

## Abstract

**Background:**

Radiomics analysis of ^18^F-FDG PET/CT enables extraction of high-dimensional quantitative features that may serve as imaging biomarkers for tumor biology. In particular, in non-small cell lung cancer (NSCLC), such features could potentially predict immune checkpoint biomarker status, such as PD-L1 expression, and histological subtype without the need for invasive tissue sampling.

**Methods:**

We retrospectively analyzed pre-treatment ^18^F-FDG PET/CT scans of 115 patients with histologically confirmed NSCLC (adenocarcinoma or squamous cell carcinoma) and known PD-L1 status. Radiomic features were extracted from primary tumors and combined with basic clinical variables (“*naïve features*”). Principal component analysis (PCA) and hierarchical clustering were performed to assess separability between the selected classes representing either PD-L1 subtype or histology. Multiple machine learning classifiers, including random forest (RF), were trained using radiomic-only, naïve-only, and combined feature sets. Model performance was evaluated by area under the receiver operating characteristic curve (AUC-ROC).

**Results:**

For PD-L1 prediction, the RF model trained on radiomic features achieved the highest accuracy (AUC-ROC = 0.83, 95% CI = [0.75 - 0.91]), outperforming the combined feature model (AUC-ROC = 0.81, 95% CI = [0.73 - 0.89]) and naïve-only generalized linear and RF models (AUC-ROC ~ 0.5). Heatmap analysis of selected radiomic features showed significant clustering of PD-L1-positive and -negative tumors (Fisher’s exact *p* = 7 × 10^− 4^). For histological subtype classification, the best-performing RF model achieved an AUC-ROC of 0.76 using radiomic features. Although PCA demonstrated partial separation between adenocarcinoma and squamous cell carcinoma (PC1 = 38%, PC2 = 21%), the hierarchical clustering led to the significant class separation (*p* = 0.0234).

**Conclusions:**

Our pilot project indicated that ^18^F-FDG PET/CT radiomics enables accurate non-invasive prediction of PD-L1 expression and provides moderate discrimination between major NSCLC histological subtypes. Clinical variables alone have negligible predictive value for PD-L1 status. Radiomic models may complement histopathology, particularly when tissue sampling is limited or repeated biomarker assessment is required. Prospective, multi-center validation is needed to confirm generalizability and facilitate clinical translation.

**Supplementary Information:**

The online version contains supplementary material available at 10.1186/s12967-026-08029-w.

## Purpose

Lung cancer remains one of the leading causes of cancer-related mortality worldwide, with non-small cell lung cancer (NSCLC) accounting for approximately 85% of cases [1]. In recent years, the introduction of immune checkpoint inhibitors (ICIs), particularly those targeting the programmed death-1 (PD-1) and programmed death-ligand 1 (PD-L1) axis, has significantly transformed the therapeutic landscape for patients with advanced NSCLC. Accurate characterization of NSCLC at the time of diagnosis, including PD-L1 expression status and histological subtype, is essential for treatment planning and prognostic assessment [2]. PD-L1 expression assessed by immunohistochemistry (IHC) on biopsy samples is the primary biomarker used to guide the use of ICIs [3]. In current clinical practice, both histological classification and PD-L1 evaluation rely on tissue biopsy. However, this method presents several limitations. It is invasive, prone to sampling errors due to tumor heterogeneity (both at intra- and intertumoral level), and does not allow for dynamic monitoring of PD-L1 status throughout disease progression. Additionally, the limited availability of tissue samples in some patients restricts the feasibility of repeated testing [4, 5]. These limitations have stimulated growing interest in non-invasive, imaging-based approaches that could provide complementary or surrogate information to support diagnostic and therapeutic decisions.

Radiomics, a rapidly evolving field leveraging the extraction of high-dimensional quantitative features from standard-of-care imaging, has emerged as a promising non-invasive approach to capture intratumoral heterogeneity and underlying tumor biology [6]. ^18^F-fluorodeoxyglucose positron emission tomography/computed tomography (^18^F-FDG PET/CT), widely used in lung cancer staging, combines metabolic and anatomical information, offering a comprehensive overview of tumor burden and metabolic activity. Beyond its established role in tumor detection and treatment response assessment, ^18^F-FDG PET/CT imaging may also capture subtle imaging patterns reflecting the biological behavior of the tumor [7]. Quantitative analysis of these patterns through radiomics allows for the extraction of high-dimensional data that are not accessible through visual image interpretation alone. Recent guideline by the European Association of Nuclear Medicine and the Society of Nuclear Medicine and Molecular Imaging, highlights the potential of radiomics in nuclear medicine, provided that standardized acquisition and segmentation are applied [8]. It is hypothesized that such radiomic features, derived from routine ^18^F-FDG PET/CT scans, may correlate with key tumor characteristics, including PD-L1 expression and histological subtype, which are typically determined by invasive tissue sampling [9]. The concept of using PET/CT-based radiomics as a “digital biopsy” seeks to utilize this potential by providing clinically relevant information about tumor phenotype in a non-invasive, repeatable, and cost-effective manner. Unlike conventional biopsies, which are subject to sampling bias and limited by tumor heterogeneity, radiomics enables whole-lesion analysis, potentially offering a more comprehensive and reproducible assessment [10]. Although preliminary studies have explored the feasibility of radiomics-based prediction models, further research is needed to systematically evaluate their clinical utility, particularly in the context of simultaneous prediction of PD-L1 status and histological subtype from a single imaging modality.

The aim of this pilot study is to develop and evaluate a non-invasive model for the prediction of PD-L1 expression and histological subtype in patients with NSCLC using ^18^F-FDG PET/CT. We hypothesized that image-derived features reflecting tumor structure and texture provide incremental predictive value over clinical parameters and could complement histopathological assessment, particularly in cases with limited or unavailable tissue. Confirming these hypotheses would support the role of ^18^F-FDG PET/CT radiomics as a clinically relevant surrogate for tissue-based biomarker assessment and a potential tool for individualized treatment planning and improved patient stratification.

## Methods

### Patient selection

A retrospective analysis was conducted on a cohort of consecutive patients diagnosed with biopsy-confirmed non-small cell lung cancer (NSCLC), predominantly at advanced clinical stages (stage III or IV). All patients were diagnosed at the Department of Pathomorphology, Medical University of Gdańsk, Poland between July 2018 and October 2021. Corresponding ^18^F-FDG PET/CT imaging was performed at the Department of Nuclear Medicine of the same institution. The PD-L1 classification task was well balanced (57 PD-L1–negative vs 58 PD-L1–positive cases), and histological subtypes were also reasonably balanced, reducing the risk of class imbalance-driven bias. Random forest-based models were used due to their robustness to correlated predictors and non-linear feature interactions.

**Fig. 1:**
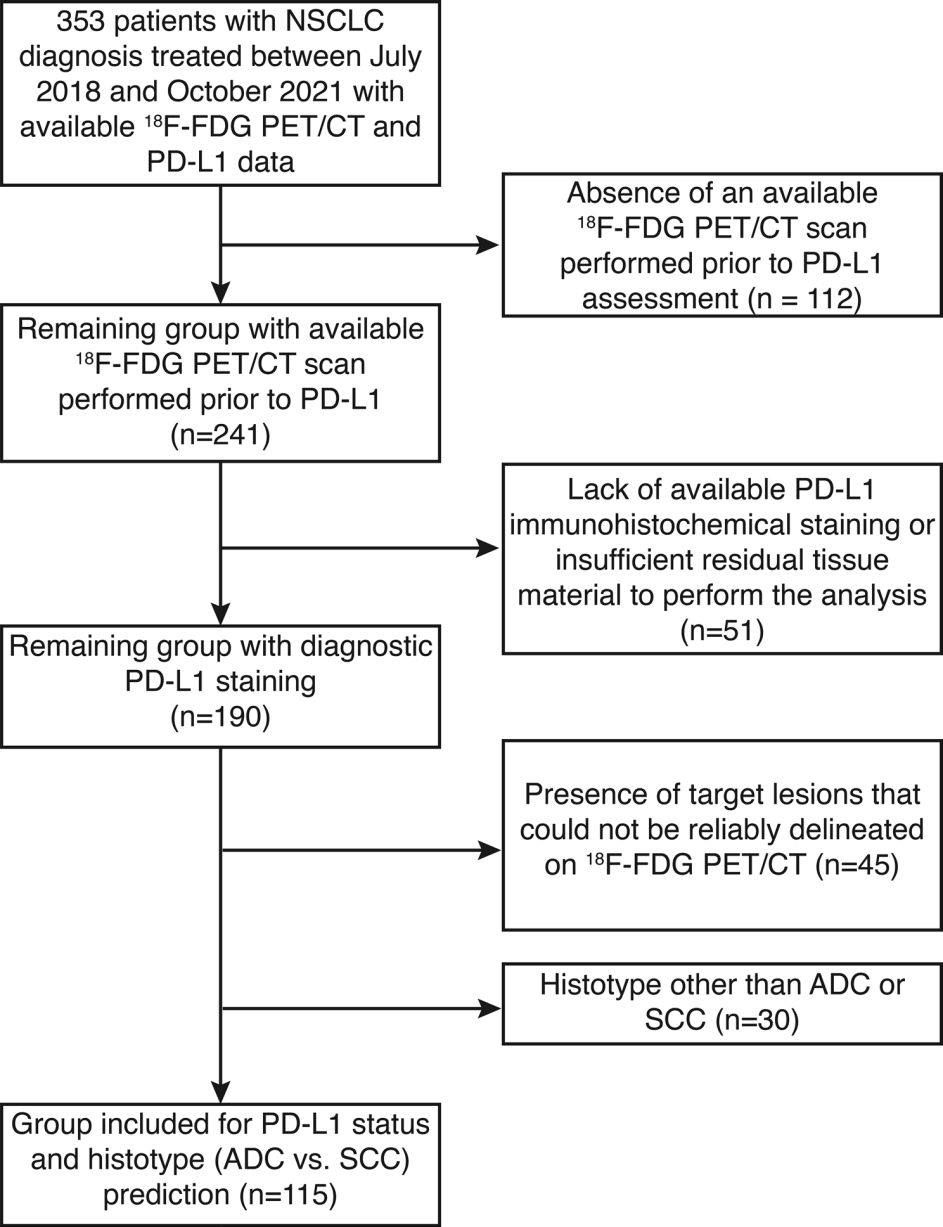
Flowchart presenting patient selection and exclusion criteria. Adenocarcinoma - ADC, squamous cell carcinoma - SCC, PD-L1 - programmed death-ligand 1.

From the initial cohort of 353 patients, several exclusion criteria were applied to refine the study population (Fig. 1, Supplementary Figs. 1–2). All exclusion criteria were defined a priori and were related to data availability or technical feasibility of radiomic analysis. No patients were excluded based on PD-L1 expression, clinical stage, or imaging-derived characteristics. Patients were excluded if they met any of the following conditions (1): absence of an available ^18^F-FDG PET/CT scan performed prior to PD-L1 assessment (2); lack of available PD-L1 immunohistochemical staining or insufficient residual tissue material to perform the analysis (3); presence of target lesions that could not be reliably delineated on ^18^F-FDG PET/CT imaging or (4) histotype other than adenocarcinoma (ADC) or squamous cell carcinoma (SCC).

For each patient, relevant clinical and pathological data were collected, including age and sex at diagnosis, histological subtype of NSCLC, PD-L1 expression status, and clinical stage according to the 8^th^ edition of the American Joint Committee on Cancer (AJCC) staging system [11]. PD-L1 expression was categorized according to tumor proportion score (TPS) thresholds of <1% (considered negative), 1–49%, and ≥50% (both considered positive).

### Acquisition parameters

The patients were injected with an activity of 3.3 MBq of ^18^F-FDG per kg of body weight. Imaging was performed solely with a 40-detector PET/CT scanner (Siemens Biograph mCT 40, Siemens Healthineers, Erlangen, Germany) after an uptake period of approximately 60 minutes. PET emission data were acquired with 6–8 bed positions (depending on patients’ height) from the base of the skull to the proximal thighs (2 minutes emission time per bed position for <90 kg of body weight, 3 minutes for 90-120 kg, and 4 minutes for ≥120 kg patients). Subsequently, a monophasic low-dose CT scan without contrast media was performed (CARE Dose 4D, 160 mAs, 120 kV, 512 × 512 matrix, 2 mm slice thickness, slice collimation 64 × 0.6 mm, pitch index: 1.25). All PET emission data were reconstructed with an iterative algorithm (TrueX+TOF, ultra HD-PET, 21 subsets, 2 iterations, Gaussian filtering: 4.5 mm, Zoom 1.0, 171 × 200 × 200 matrix, axial resolution: 5 mm, in-plane resolution: 4.07 × 4.07 mm^2^) using dedicated manufacturer software (syngo MI.PET/CT, Siemens Healthineers, Erlangen, Germany). No adverse events including allergic reactions were observed after administration of radiotracer. SUV quantification was based on total body weight.

### Detection of PD-L1 expression

The PD-L1 expression level was determined in the primary tumor. Biopsy material was fixed in 10% neutral buffered formalin, processed for routine histopathology and embedded in paraffin. Tissue sections were stained with PD-L1 IHC 22C3 pharmDx assay (clone 22C3). PD-L1 expression was determined as per tumor proportion score (TPS) and was dichotomized into negative (<1%) and positive (≥1%) to reflect a clinically meaningful decision threshold and to ensure model stability in a moderate-sized cohort.

### Segmentation and image conversion

All tumor segmentations were performed using a standardized semi-automatic workflow followed by expert manual refinement by experienced radiation oncologist and nuclear medicine specialists. The same software, segmentation protocol, and imaging data derived from a single PET/CT scanner were used for all cases, aiming to minimize segmentation-related variability. Formal quantitative assessment of inter-observer variability was not performed in this study. ^18^F-FDG PET/CT DICOM images were processed using Slicer 3D v. 5.2.2. PET radiomics plugin (https://github.com/QIICR/Slicer-PETDICOMExtension) was first installed and used for automatic SUV normalization and initial semiautomatic contouring of the primary tumors [12]. After that, manual refining of the contoured regions was performed by experienced radiation oncologist (BT) and nuclear medicine specialists (WC and JT) in order to include all uneven areas of the primary malignant tissue including necrotic regions, occasionally skipped by the software. In cases of particularly challenging delineation of the tumors on ^18^F-FDG PET/CT images fused PET/CT images and/or CT images alone were used to navigate the contouring. All target lesion volumes were larger than 5 ml. After segmentation, each individual session was saved to produce NRRD type files of the original image (*image.nrrd*) along with the accompanying segmentation file (*segmentation.seg.nrrd*). All the above steps were performed by experienced bioinformatician (MJ).

### Radiomic features extraction

To extract radiomics features from ^18^F-FDG PET/CT images we used the PyRadiomics package, version v3.0.1 (https://pyradiomics.readthedocs.io/) [13]. Extraction was performed in a batch mode for all patients and their corresponding images and masks. A batch input file consisted of three columns, i.e. patient ID, Image, and Mask. To get an extended set of radiomics features, beside the default 120 (https://pyradiomics.readthedocs.io/en/latest/features.html), we used a custom parameter file (param.yaml). By enabling various transformations (log-sigma-1–0-mm-3D, log-sigma-3–0-mm-3D, log-sigma-5–0-mm-3D, wavelet-LH, wavelet-HL, wavelet-HH, wavelet-LL, square, squareroot, logarithm, exponential) the number of extracted features was increased to 1120. The above calculations were made in Python (version 3.10.12). The features that were used met the definition described by the Imaging Biomarker Standardization Initiative [14]. All radiomic features were extracted using a fixed PyRadiomics configuration, with preprocessing steps, enabled feature classes, and image transformations fully specified in a YAML parameter file provided in the Supplementary Materials and the project’s GitHub repository.

The generated features were imported into R (version 4.1.2) [15]. Prior to any downstream calculations columns/features with zero variability were removed, similar to any diagnostic features, except of CenterOfMass and CenterOfMassIndex. The latter two columns were split into new individual numeric columns, as originally, they are stored in triplets separated by a comma. We next added some extra columns that represent patient-based features, such as histological type of cancer, sex, SUV_max_ value, as well as information on the primary tumor contouring expert (BT, WC or JT). If a feature was non-numeric, e.g. sex, they were stored by adding an extra column per each value of the feature and encoding the membership by 0 or 1 (e.g. sex.Male, sex.Female). Lastly, prior to modeling the outcome value for the predictive model was defined by binning the PD-L1 expression values into two major categories, namely negative (TPS < 1%) and positive (TPS ≥ 1%).

### Statistical analysis

For both analyses, i.e. PD-L1 status or histological subtype, the same patient cohort was used. From the initial set of 1107 radiomic features, 236 were removed due to a coefficient of variation (standard deviation divided by mean) below 0.2. The remaining 871 radiomic features were then analyzed using the Wilcoxon test to compare differences between patient groups stratified by PD-L1 levels or histological types separately. In addition to conventional radiomic features, we also considered their pairwise interactions, defined as the product of two features. Interaction terms were explored to capture potential non-linear relationships between radiomic features, but were treated as candidate features subject to the same supervised selection and dimensionality reduction as original features. To avoid computational overload, we restricted this procedure to features that showed preliminary discriminatory power in univariate testing. For interaction analysis, only features that met predefined significance thresholds in univariate testing were selected as “seed” variables. Each of these features was then multiplied with every other available feature to generate product terms. Interaction terms were retained only if their Wilcoxon *p*-value was at least five times lower than the applied threshold. To achieve feature sets of comparable size between the two analyses, different thresholds were applied: *p* < 0.05 for PD-L1 classification and *p* < 0.01 for histology classification. Different univariate significance thresholds were applied for PD-L1 and histological subtype analyses to obtain feature sets of comparable size and stability, reflecting differences in class separability between the two tasks, as supported by sensitivity analyses reported in the Supplementary Table 3. As a result, 2340 features (original radiomic features and selected products) were included in the PD-L1 analysis, and 2635 in the histology analysis. These expanded feature sets were subsequently subjected to supervised feature selection using the Monte Carlo Feature Selection (MCFS) algorithm [16]. The MCFS was performed globally on the development cohort prior to cross-validation to identify compact, biologically informative feature sets in this pilot, hypothesis-generating study. This approach was not intended as a strict nested feature selection strategy. Briefly, MCFS is a supervised feature selection method that randomly constructs multiple tree classifiers from the original training dataset and assigns importance to features based on their role in classification. We ran the MCFS using the *mcfs* function from the rmcfs R package (version 1.3.5) with the following settings: *adjucutoffPermutations* = 100, *featureFreq* = 250, *mode* = 2, *balance* = 1, *finalCV* = FALSE, *finalRuleset* = FALSE, *spilts* = 20, and *threadsNumber* = 6. All other parameters were set to their defaults. This procedure reduced the number of features to 35 and 68 for PD-L1 status and histology analyses, respectively.

### Machine learning classification

All models presented here were built using the ‘caret’ package (version 6.0.86) with 10-fold cross-validation repeated 20 times [17]. The *summaryFunction* parameter was set to *twoClassSummary*. In total, one linear classifier and eight different machine learning classifiers were employed (specified using the method parameter in the train function): generalized linear model (*glm*), linear discriminant analysis (*lda*), k-nearest neighbors (*knn*), support vector machine with radial basis function kernel (*svmRadial*), random forests (*rf*), PAMR (*pam*), neural networks (*nnet*), bagging (*treebag*), and recursive partitioning (*rpart*). Clinical (‘naïve’) features included age, sex, histological subtype (for PD-L1 prediction models), and SUVmax of the primary tumor. Individual T, N, and M categories were reported descriptively but were not included in the modelling to avoid redundancy and collinearity with overall stage. Smoking status was not included due to incomplete availability in the retrospective dataset. For each classifier type, the optimal model was selected based on the receiver operating characteristic (ROC metric), and the maximum number of iterations was set to 2000 (*maxit* parameter). The models were assessed based on the average values over the obtained results. The ROC curves were plotted using the final models and the entire dataset. Random forest was selected as the reference classifier due to its robust performance in high-dimensional feature spaces, resistance to multicollinearity, and stable performance across repeated cross-validation.

### External validation cohort

An independent external validation cohort consisted of 82 patients with biopsy-confirmed NSCLC treated at the Medical University of Łódź, Poland, during the same study period as the development cohort. All patients underwent pre-treatment ^18^ F-FDG PET/CT imaging, and only cases fulfilling the same inclusion and exclusion criteria as in the development cohort were included.

In the external validation cohort, pre-treatment ^18^ F-FDG PET/CT examinations were performed using a different PET/CT scanner model than in the development cohort (Biograph mCT 128, Siemens Healthineers, Erlangen, Germany). Imaging was conducted according to the local clinical protocol after standard patient preparation and radiotracer administration. Image acquisition was typically initiated approximately 60 minutes after tracer injection, with imaging performed in a standard whole-body field-of-view extending from the skull base to the proximal thighs.

PET emission data were reconstructed using a three-dimensional ordered-subsets expectation maximization algorithm (3D-OSEM; 4 iterations, 8 subsets) with point-spread-function modeling (TrueX). PET images were reconstructed with an in-plane pixel spacing of approximately 4.07 × 4.07 mm^2^ and a slice thickness of 1.5 mm. CT-based attenuation correction was applied.

The CT component of the PET/CT examination was performed as a non-contrast, low-dose scan according to routine clinical protocols and used for attenuation correction and anatomical reference. CT images were reconstructed on a 512 × 512 matrix with an in-plane pixel spacing of approximately 0.98 × 0.98 mm^2^ and a slice thickness of 1.5 mm, using a standard soft-tissue reconstruction kernel. Automatic tube current modulation was applied. PET images were converted to standardized uptake values normalized to body weight.

### Code availability

Code used to process the data is available at https://github.com/jakubmie/NSCLC_PDL1_radiomics the matrix of the raw extracted radiomics features can be shared upon reasonable request. The complete PyRadiomics YAML configuration file used for feature extraction is provided in the Supplementary Materials.

### Ethics statement

This study was approved by the Bioethical Committee of the Medical University of Gdańsk (NKBBN/688/2022) and performed under relevant guidelines and regulations, including the Helsinki Declaration of 1975. Due to the retrospective character of the study, patient consent was not required.

## Results

### Group characteristics

**Fig. 2:**
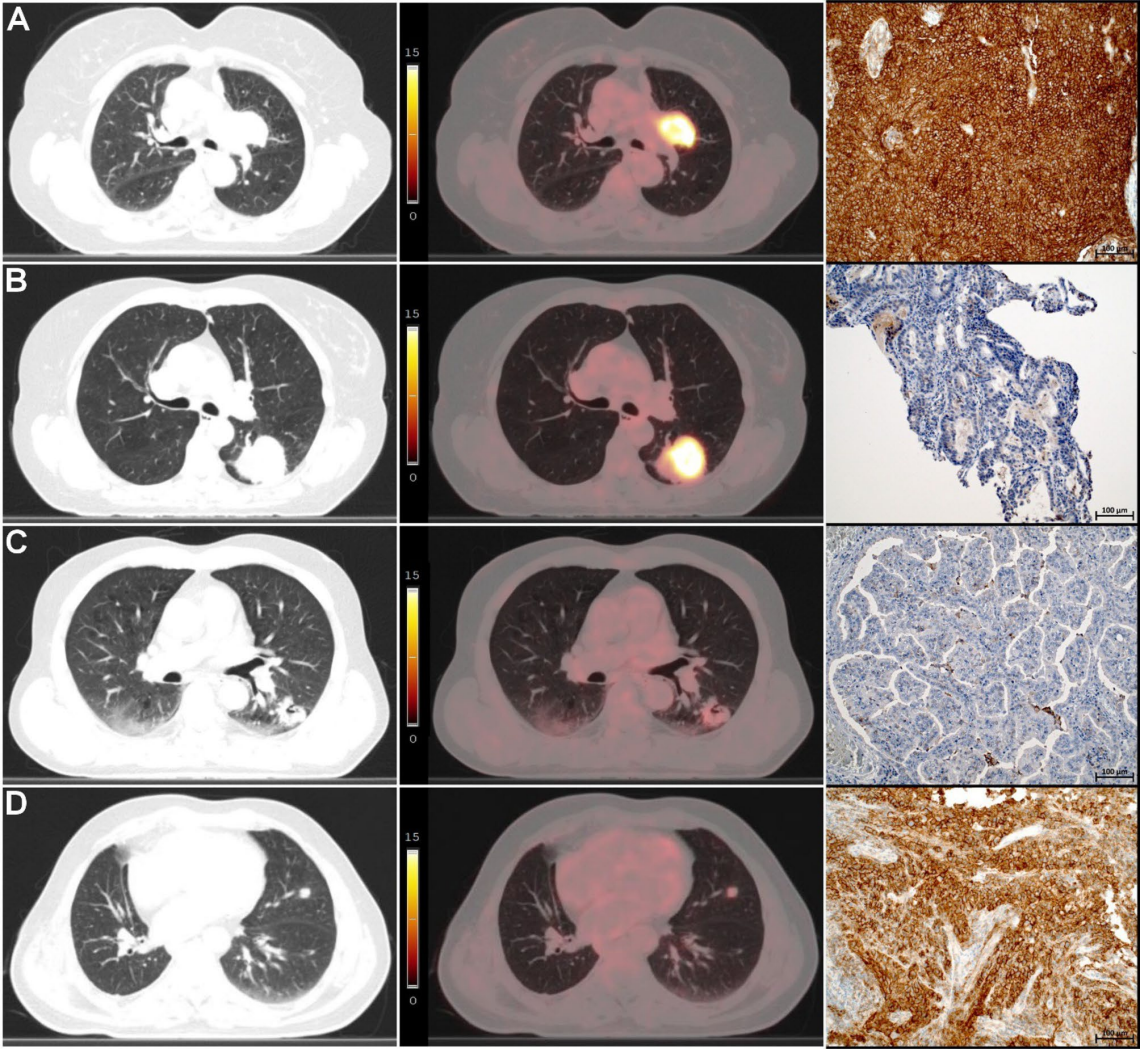
Examples of primary lung cancers demonstrating various levels of metabolic activity (SUVmax) vs. PD-L1 expression (TPS). Row A - SUV_max_ =26.5, TPS = 95%; row B - SUV_max_ =21.6, TPS < 1%; row C - SUV_max_ =4.4, TPS < 1%; row D - SUV_max_ =3.1, TPS = 85%

The final cohort available for analyses encompassed 115 patients, was well balanced, and is characterized in Table 1. Briefly, 49 (43%) of patients were female, with an overall median age of 70 years (IQR 65 - 76). Samples for all patients were taken from the primary lung lesion. Adenocarcinoma (ADC) was reported in 51 patients (44%) while squamous cell carcinoma (SCC) in 64 patients (56%). In the entire cohort, 57 (50%) patients were negative (<1%) for PD-L1 IHC, samples from 36 patients (31%) showed PD-L1 expression in 1–49% of cells and in 22 patients (19%) in ≥50%. The comparison of PD-L1 expression distribution among histotypes (ADC vs. SCC) did not demonstrate statistically significant differences (*p* = 0.181). Examples of primary lung cancers demonstrating various levels of metabolic activity and PD-L1 expression are presented in Fig. 2.

**Table 1:** Basic clinical features of patients with negative and positive PD-L1 expression

Factor (feature)	PD-L1 negative (*n* = 57)	PD-L1 positive (*n* = 58)	p-value
Age [years] (median and IQR)	70 (65–76)	69 (66–75)	0.730
Sex * Male* * female*	34 23	32 26	0.627
T stage * 1* * 2* * 3* * 4*	7 8 16 26	11 6 14 27	0.257
N stage * 0* * 1* * 2* * 3*	11 9 25 12	16 8 23 11	0.776
M stage * 0* * 1*	37 20	41 17	0.507
Clinical stage * I* * II* * III* * IV*	7 5 25 20	4 7 30 17	0.533
Histological type * Adenocarcinoma* * squamous*	30 27	21 37	0.076
SUV_max_ (mean ± SD)	22.4 ± 13.2	19.0 ± 8.3	0.103

### Prediction of PD-L1 status

**Fig. 3:**
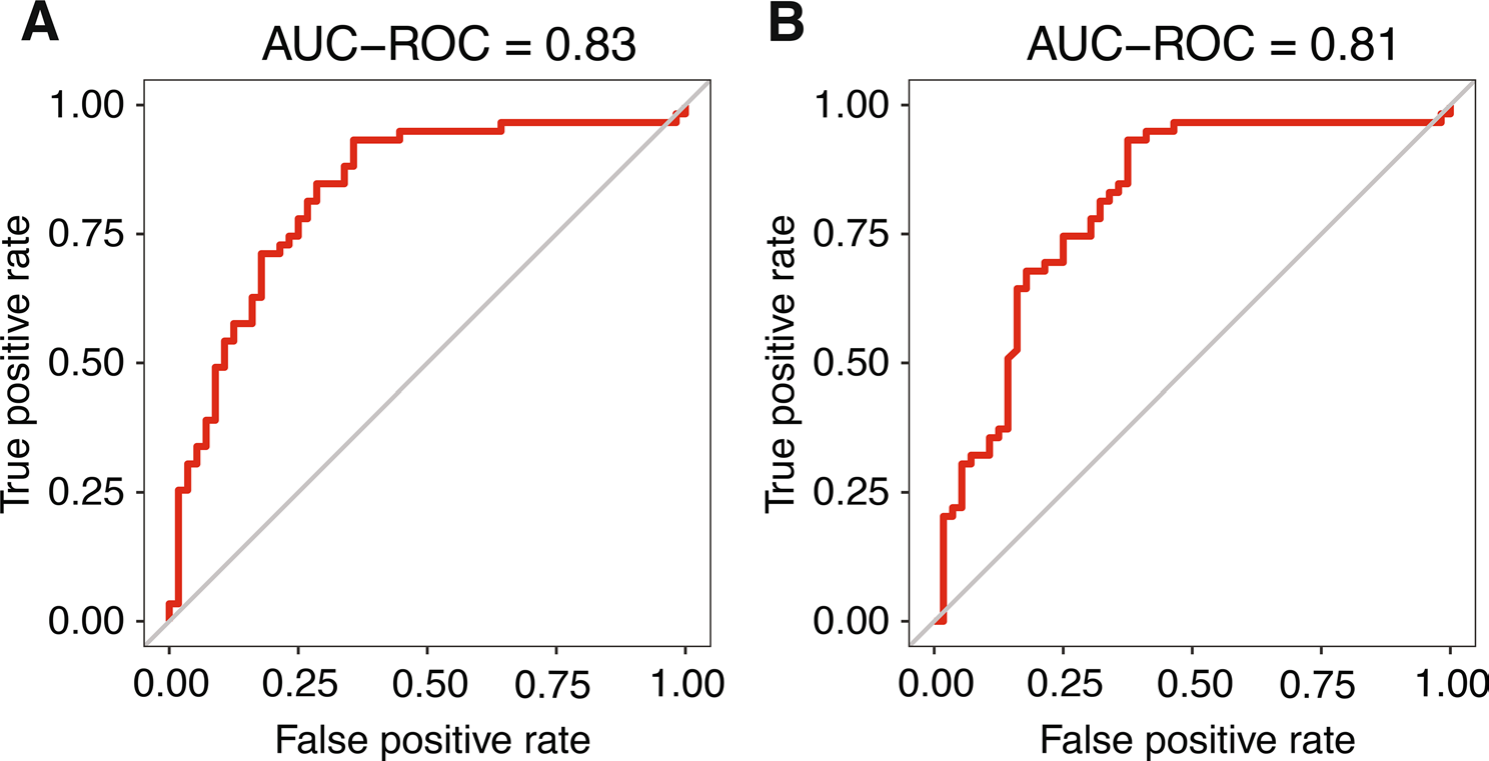
Receiver operating characteristic (ROC) curves for PD-L1 classification using random forest models trained on (**A**) radiomic features only and (**B**) combined radiomic and clinical (naïve) features

Initial univariate analysis showed no statistically significant association between SUVmax and PD-L1 expression (Table 1). When only basic clinical variables (naïve features) were used for model training, predictive performance was poor, with AUC-ROC values close to random classification (glm: 0.50; RF: 0.45, Supplementary Fig. 1A and 1B). These results indicate that clinical variables alone carried negligible predictive value for PD-L1 expression compared to models leveraging radiomic data. PCA based solely on radiomic features revealed a partial separation of PD-L1 positive and negative tumors along the first two principal components (PC1 = 38%, PC2 = 21%, Supplementary Fig. 2). When comparing different machine learning classifiers, the best performance for PD-L1 classification was achieved by the random forest (RF) model trained exclusively on radiomic features, with an AUC-ROC of 0.83 (Fig. 3A). Combining radiomic and naïve features resulted in a slightly lower AUC of 0.81 (Fig. 3B).

**Fig. 4:**
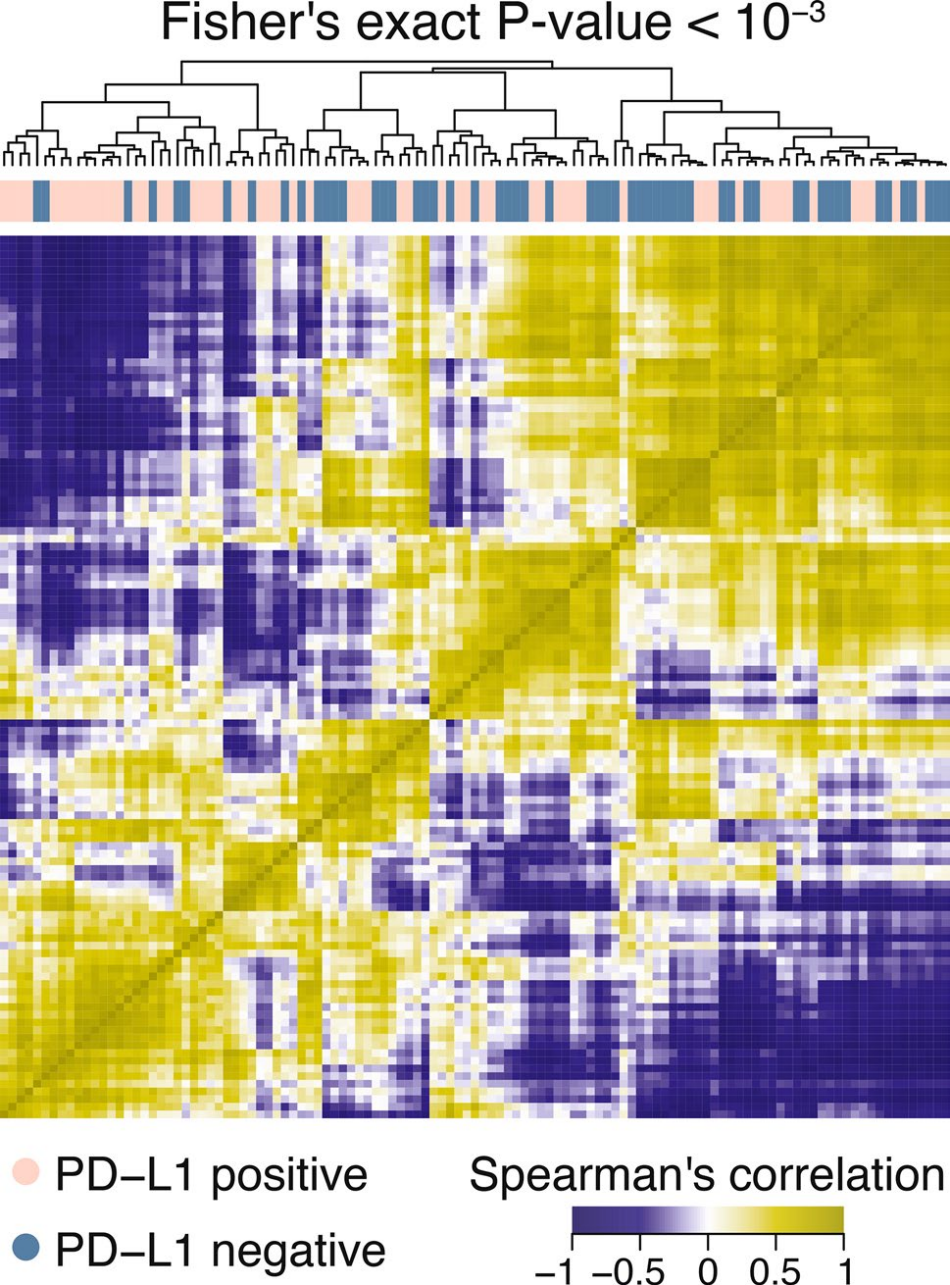
Heatmap showing Spearman’s correlation coefficients computed for the selected radiomic features in the analyzed cohort. The blue and pink bars above the heatmap indicate PD-L1 status. Hierarchical clustering revealed a separation into two main clusters, which were significantly associated with PD-L1 expression. (Fisher’s exact test p = 7×10⁻⁴)

Performance comparison across all evaluated classifiers is shown in Supplementary table 4 and Supplementary Fig. 3. The heatmap of selected radiomic features demonstrated distinct clustering of PD-L1 positive and negative tumors (Fisher’s exact test *p* = 7 × 10^− 4^), with multiple features showing strong correlation patterns (Fig. 4)

### Prediction of histological subtype

**Fig. 5:**
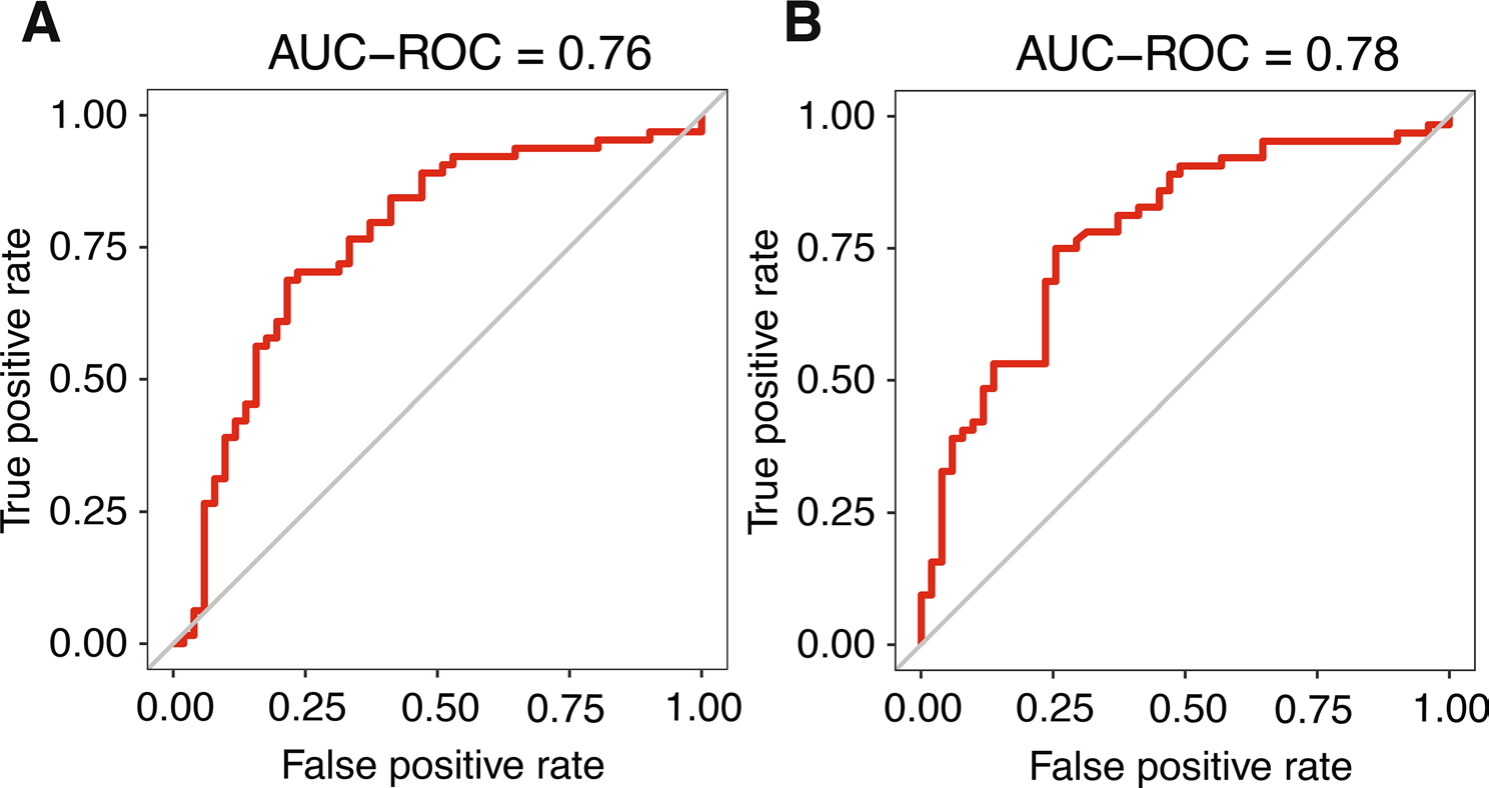
Receiver operating characteristic (ROC) curves for histotype classification using random forest models trained on (**A**) radiomic features only and (**B**) combined radiomic and clinical (naïve) features

When only basic clinical variables (naïve features) were used for model training, the predictive performance was limited. The generalized linear model achieved an AUC-ROC of 0.63, while the random forest model reached 0.70 (Supplementary Fig. 4A-B). PCA based on radiomic features allowed partial separation of adenocarcinoma and squamous cell carcinoma cases (PC1 = 38%, PC2 = 21%, Supplementary Fig. 4C). The RF classifier trained on radiomic features achieved an AUC-ROC of 0.76 for histotype prediction (Fig. 5A). Combining radiomic and naïve features led to a slight improvement in performance, with an AUC-ROC of 0.78 (Fig. 5B).

**Fig. 6:**
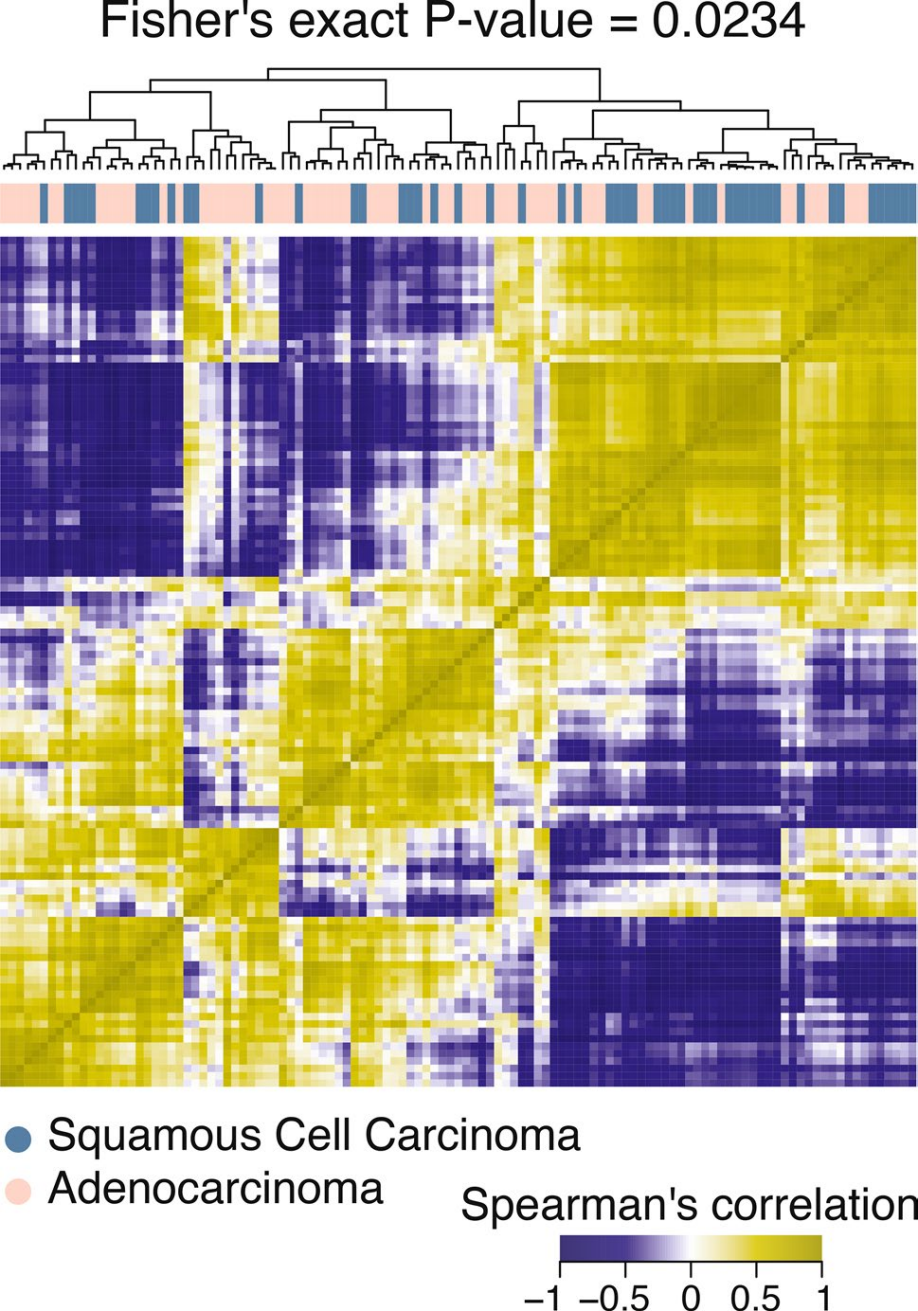
Heatmap of selected radiomic features demonstrated distinct clustering patterns between histotypes

Performance comparison across all tested classifiers is shown in Supplementary table 5 and Supplementary Fig. 5. The heatmap of selected radiomic features demonstrated distinct clustering patterns between histotypes (Fisher’s exact test *p*=0.0234; Fig. 6)

### External validation

Independent external validation was performed using a cohort of 82 NSCLC patients from a different clinical center. Imaging was acquired on different PET/CT scanners and tumor segmentation was performed by independent clinicians, while PD-L1 expression and histological subtype were assessed using the same pathological protocols as in the development cohort (Supplementary Fig. 6). The external validation cohort was used exclusively for out-of-sample performance assessment and did not inform model selection, feature selection, or decision threshold optimization.

The final random forest model, trained on the development cohort, was applied to the external dataset without re-training. For PD-L1 prediction, the model achieved an AUC of 0.75, with an accuracy of 0.76, sensitivity of 0.62, and specificity of 0.80. For histological subtype classification, the corresponding AUC was 0.74, with an accuracy of 0.71, sensitivity of 0.62, and specificity of 0.74. Confusion matrices for the external validation are provided in the Supplementary Table 7.

## Discussion

This study should be interpreted as a pilot, hypothesis-generating investigation aimed at evaluating the feasibility and potential clinical relevance of ^18^ F-FDG PET/CT radiomics for non-invasive assessment of PD-L1 status and histological subtype in NSCLC. We demonstrated that ^18^F-FDG PET/CT radiomics can provide clinically relevant information, outperforming routine clinical variables, for both PD-L1 status and histological subtype prediction in NSCLC. Importantly, we observed higher accuracy for PD-L1 status classification when using radiomic features compared to clinical variables alone, and moderate performance for adenocarcinoma vs. squamous cell carcinoma discrimination. The added value of imaging-derived features over conventional parameters supports the growing concept of a “*digital biopsy*” for molecular characterization of lung cancer [10, 18].

Although PD-L1 expression is a continuous variable and higher TPS levels may carry distinct therapeutic implications, the present analysis focused on dichotomous classification to address a clinically relevant question: whether imaging-derived features can identify tumors with any PD-L1 expression in the absence of sufficient tissue. Extension of this approach to multi-class or continuous PD-L1 prediction would require larger, dedicated datasets. Our results for PD-L1 prediction align with the expanding body of evidence supporting radiomics-based biomarker discovery in NSCLC. Li et al. recently developed a deep learning radiomics fusion model that achieved superior performance (AUC = 0.910) for PD-L1 prediction, demonstrating that combining traditional radiomics with deep learning features enhances predictive accuracy [19]. Their fusion approach outperformed both individual radiomics (AUC = 0.785) and deep learning models (AUC = 0.867), suggesting that our observed AUC of 0.83 for radiomic-only models represents competitive performance within the current landscape. Monaco et al. evaluated ^18^F-FDG PET/CT radiomics in a multi-cohort setting and demonstrated that texture features, especially those derived from gray-level co-occurrence and run-length matrices, were significantly associated with PD-L1 expression levels [20]. They highlighted that features reflecting intratumoral heterogeneity may capture the complex immune microenvironment influencing PD-L1 upregulation. Importantly, Monaco et al. noted that SUV_max_ alone was a poor discriminator, with predictive performance improving only when higher-order textural features were incorporated, which aligns with our finding that SUV_max_ differences between PD-L1-positive and negative tumors were not statistically significant [20]. They also emphasized that radiomic signatures may be robust surrogates for PD-L1 when biopsy is unavailable, but validation across scanners and institutions remains a prerequisite for clinical use. Our AUC-ROC of 0.83 for radiomic-only models compares favorably with their reported range (0.73–0.82), likely reflecting differences in patient selection, imaging harmonization, and model optimization. In our dataset, clinical (naïve) variables alone performed poorly, with AUC values close to random classification, underscoring the need for advanced image-derived descriptors when targeting immune biomarker prediction.

For histological subtype classification, our findings are consistent with those of Li et al., who analyzed ^18^F-FDG PET/CT radiomics for differentiating adenocarcinoma from squamous cell carcinoma in NSCLC [21]. They reported that models integrating multiple radiomic features achieved AUC values in the range of 0.73–0.80, which is in line with our best-performing model (AUC-ROC 0.76 using RF). Li et al. emphasized that first-order features alone were insufficient for reliable classification and that second-order texture features, particularly those reflecting gray-level heterogeneity, contributed most to histotype discrimination [21]. However, they also noted that substantial feature overlap exists between adenocarcinoma and squamous cell carcinoma, especially in advanced-stage disease, which limits the discriminatory power of PET-based radiomics. Similar to our results, their PCA analyses showed only partial separation between histotypes, supporting the notion that histology-specific phenotypic differences may not be fully captured by PET metabolic signatures alone. Li et al. proposed that combining PET radiomics with CT-based morphological features or clinical data could enhance performance, particularly for cases with borderline metabolic profiles [21].

The clinical relevance of non-invasive PD-L1 prediction lies in addressing the limitations of biopsy, including spatial heterogeneity, temporal changes, and sampling errors. A radiomics-based surrogate could potentially complement histopathology by flagging patients likely to express PD-L1, prompting confirmatory testing or guiding treatment decisions in cases with insufficient or inaccessible tissue. In the context of histotype classification, a reliable imaging-based tool could support diagnostic workflows in situations where histological confirmation is delayed or tissue sampling is risky [9]. However, reproducibility remains a key barrier, as differences in ^18^F-FDG PET/CT acquisition, reconstruction, and feature calculation, highlighted across studies, necessitate harmonization strategies such as ComBat to enable cross-center model transferability [22–24].

Limitations of our work include the retrospective design, single-center imaging protocols, and the lack of external validation. Additionally, while the integration of clinical and radiomic features slightly decreased AUC for PD-L1 classification in our cohort, this might differ in larger datasets where complementary information could improve robustness. While our use of IBSI-compliant PyRadiomics represents best practice, imaging reproducibility across different scanners and institutions remains a fundamental challenge requiring harmonization strategies. The predominance of stage III-IV disease in our cohort reflects the epidemiological distribution of NSCLC at diagnosis, in which a substantial proportion of patients present with locally advanced or metastatic disease. Radiomic analysis of early-stage lesions is additionally limited by PET-specific factors, including partial-volume effects and increased segmentation uncertainty in small tumors; moreover, our inclusion criterion requiring primary tumor volumes > 5 mL further constrains extrapolation of the proposed models to very small, early-stage lesions. Therefore, the proposed models should not be extrapolated to screening or early-detection settings without dedicated validation.

Radiomic features are known to be sensitive to tumor segmentation. Although a standardized semi-automatic workflow with expert manual refinement was applied, formal quantitative assessment of inter-observer variability (e.g., repeated segmentations or Dice coefficient analysis) was not performed, representing a limitation of this pilot study. Future work will incorporate dedicated reproducibility analyses to systematically evaluate the impact of segmentation variability on feature stability and model performance. Because feature selection was performed globally prior to cross-validation, internal performance estimates may be optimistic. To address this limitation, model generalizability was assessed using an independent external validation cohort, in which the full modeling pipeline was applied without re-training or feature re-selection.

Although deep learning-based approaches may capture higher-order imaging representations, their reliable application typically requires substantially larger and more heterogeneous datasets than available in the present study. We therefore deliberately focused on a classical radiomics framework to maximize interpretability and robustness in this pilot setting. Nevertheless, the proposed pipeline is conceptually compatible with future integration of deep learning–derived features and CT-based radiomic descriptors, which may further improve performance in larger, multi-center cohorts.

A key strength of this study is the independent external validation performed across institutions, scanners, and clinical workflows. Despite this heterogeneity, the proposed radiomics model retained meaningful discriminatory performance, supporting its transportability beyond the development setting. These findings suggest that the observed performance is not solely driven by scanner- or site-specific effects.

## Conclusions

^18^F-FDG PET/CT radiomics enables accurate non-invasive prediction of PD-L1 expression and provides moderate discrimination between major NSCLC histological subtypes. These findings underscore the promise of radiomic approaches in augmenting current diagnostic strategies, particularly in situations where tissue sampling is challenging or repeated biomarker assessment is required. Prospective, multi-center validation and integration of radiomics with other non-invasive biomarkers (e.g., circulating tumor DNA, radiogenomics) will be key steps toward clinical translation.

## Electronic supplementary material

Below is the link to the electronic supplementary material.

Supplementary material 1
