# Supplementary material for: Non-invasive assessment of PD-L1 status and histology in non-small cell lung cancer using 18F-FDG PET/CT radiomics
Source: J Transl Med. 2026 Mar 18;24:587. doi: 10.1186/s12967-026-08029-w (PMC13112830; doi:10.1186/s12967-026-08029-w)
Supplement: Supplementary file 1 — Supplementary material 1 [file 12967_2026_8029_MOESM1_ESM.pdf]

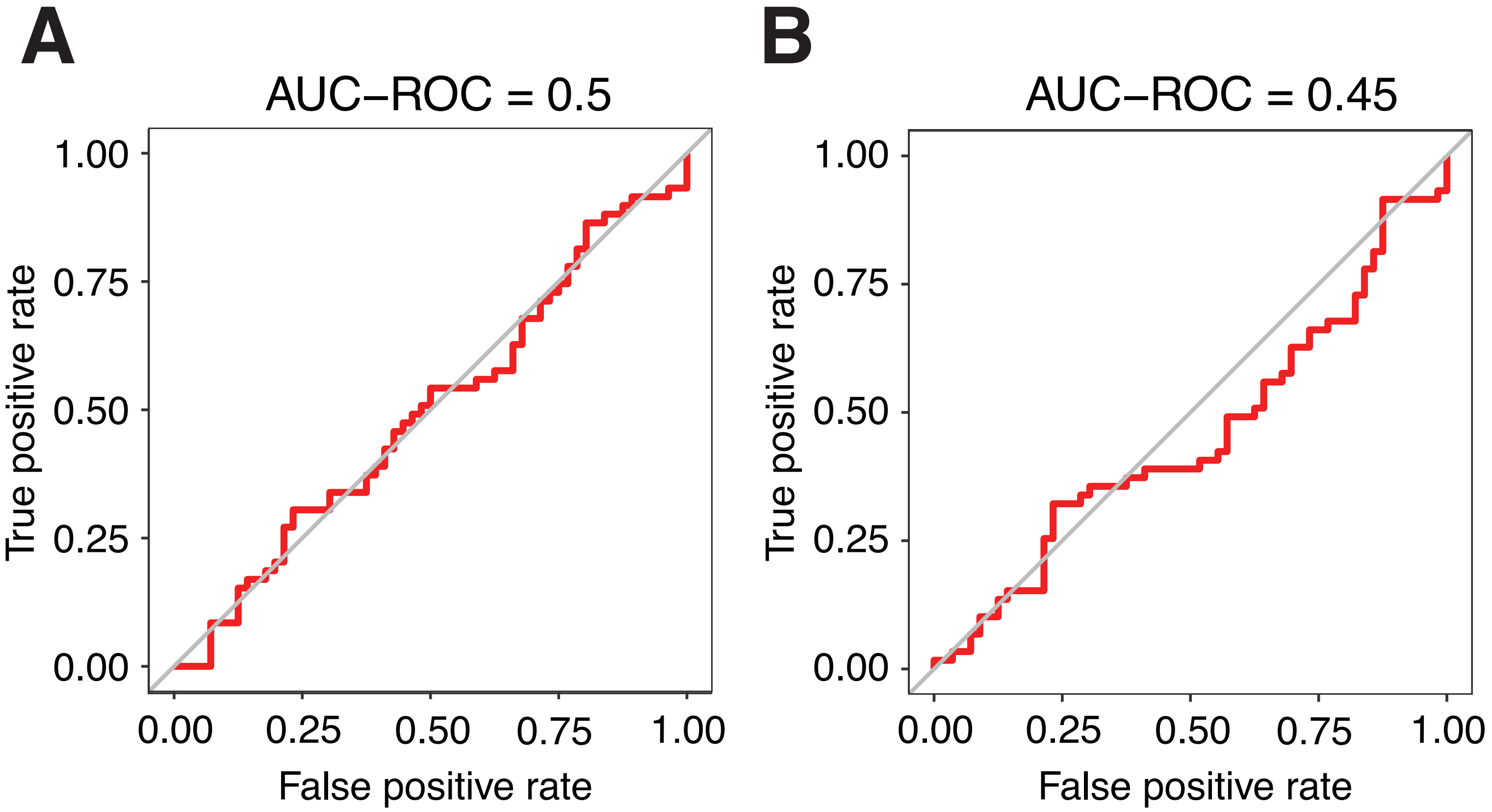

Supplementary Figure S1.
Receiver operating characteristic (ROC) curves for PD-L1 classification using generalized liner model **(A)** and random forest model **(B)** trained on clinical (naïve) features.

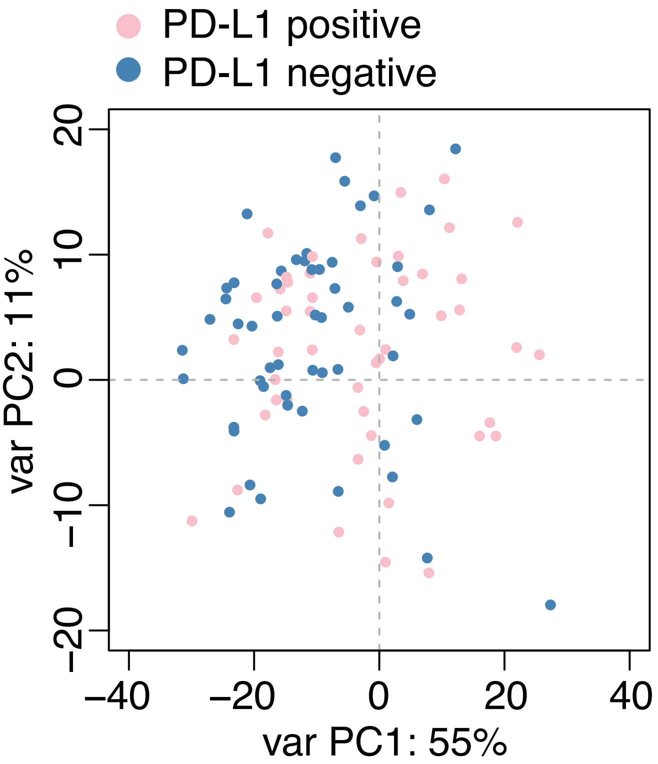

Supplementary Figure S2.
Principal Component Analysis performed on radiomic features and pairwise interactions selected for further PD-L1 classification analysis.

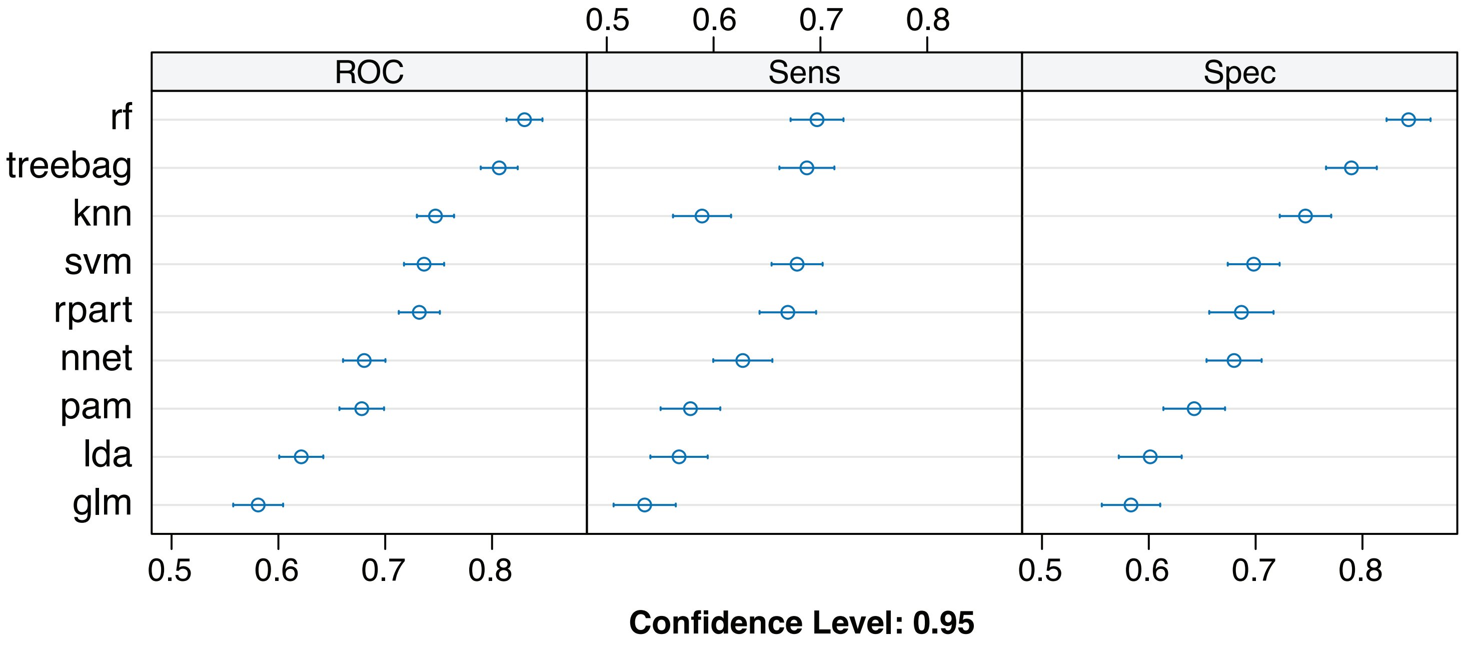

Supplementary Figure S3.
Dotplots representing comparisons of computed PD-L1 classification models

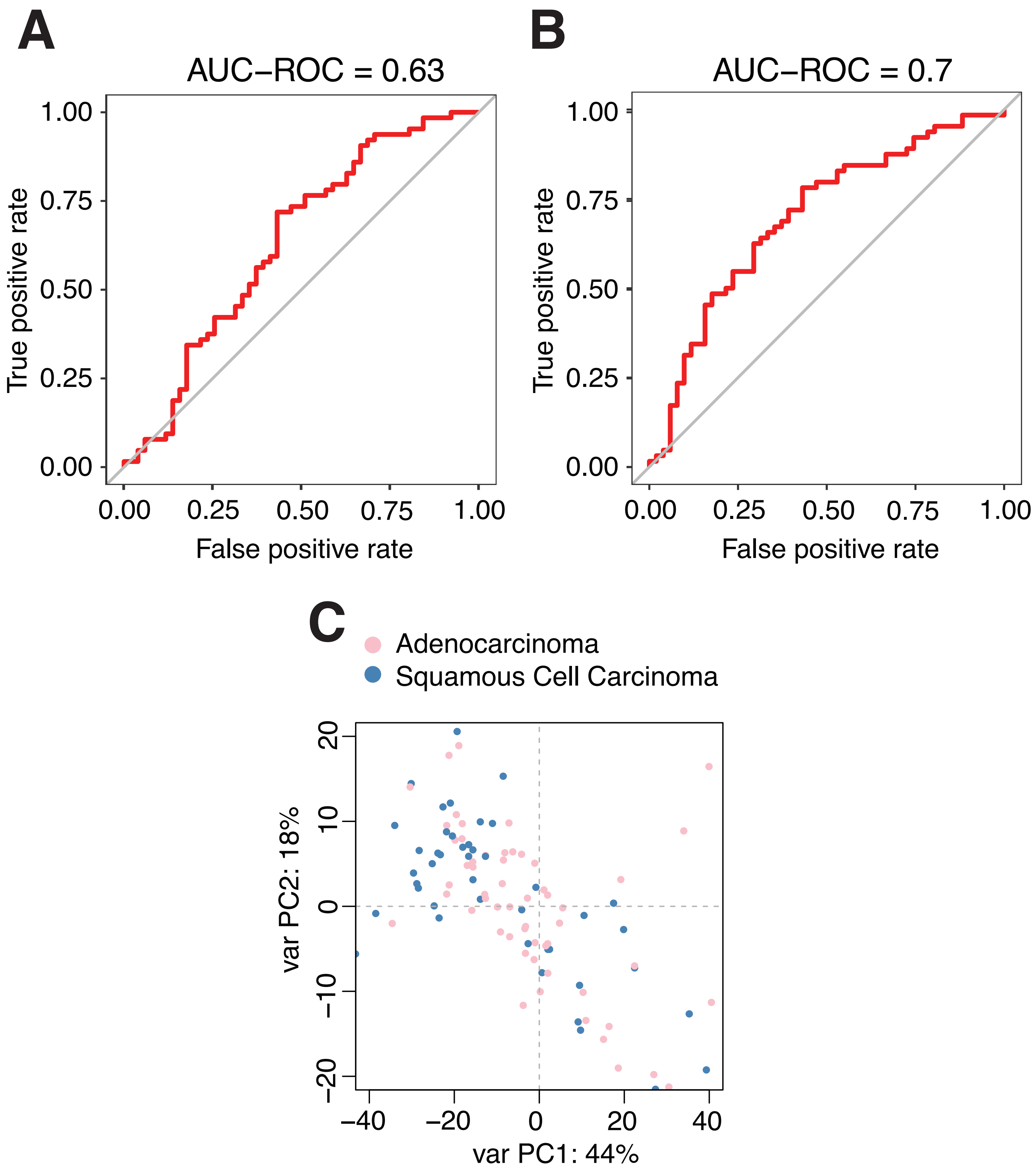

Supplementary Figure S4.
Receiver operating characteristic (ROC) curves for histological subtype classification using generalized liner model **(A)** and random forest model **(B)** trained on clinical (naïve) features. **(C)** Principal Component Analysis performed on radiomic features and pairwise interactions selected for further PD-L1 classification analysis.

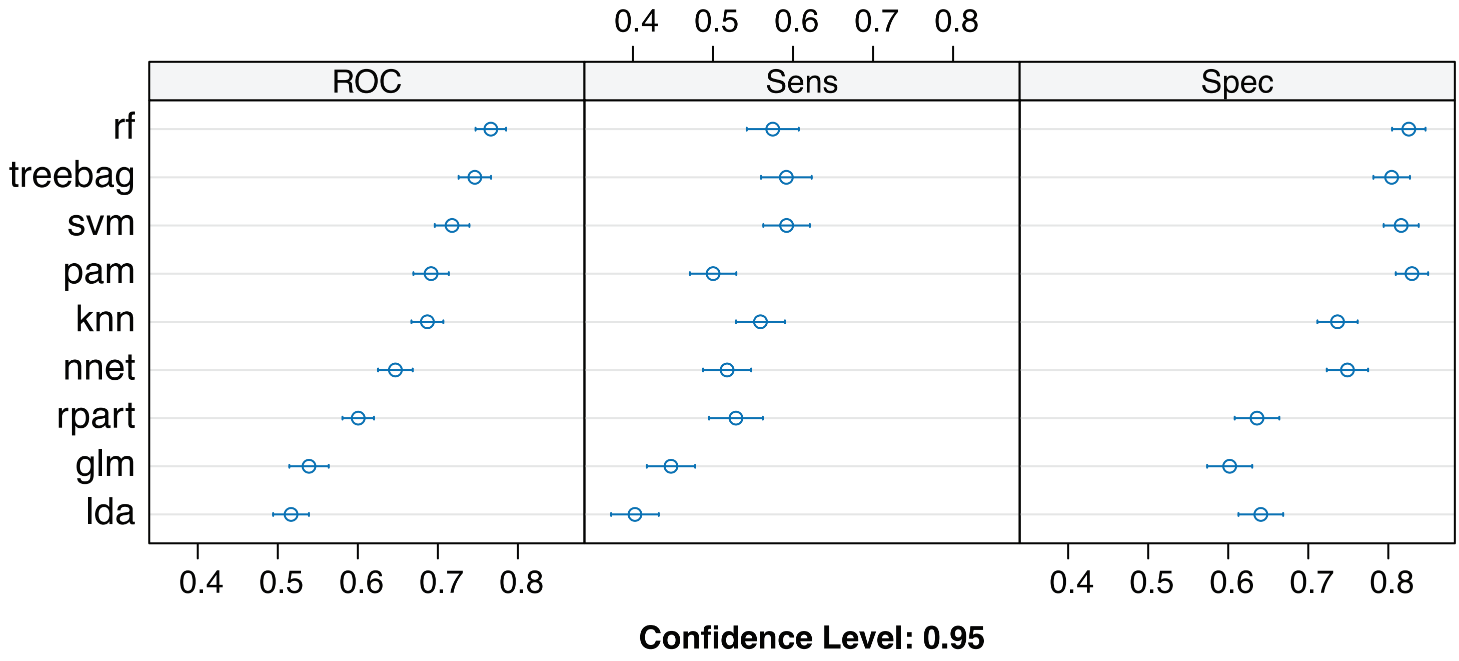

Supplementary Figure S5.
Dotplots representing comparisons of computed histological subtype classification models

Supplementary Table 1 Basic clinical features of excluded patients (n=238).

Factor (feature)	PD-L1 negative (n=117)	PD-L1 positive (n=121)	p-value*
**Age [years] (median and IQR)**	70 (66 – 76)	71 (66 – 76)	0.7781
**Sex**			0.1371
male	74	64	
female	43	57	
**Histological type**			0.5036
adenocarcinoma	51	58	
squamous	29	33	
other	37	30	

* p-value from either Mann-Whitney test for numeric values (age) and Chi-square test for categorical data

Supplementary Table 2 Comparison of basic clinical features between the included and excluded patients.

Factor (feature)	included (n=115)	excluded (n=238)	p-value*
**Age [years] (median and IQR)**	70 (66-76)	71 (66-76)	0.8998
**Sex**			
male	66	138	1
female	49	100	
**Histological type**			
adenocarcinoma	51	109	6.745e-12
squamous	64	62	
other	0	67	
**PD-L1 status**			
negative	57	117	1
positive	58	121	

* p-value from either Mann-Whitney test for numeric values (age) and Chi-square test for categorical data

Supplementary Table 3: Sensitivity analysis of univariate filtering thresholds for PD-L1 status and histological subtype prediction.
Model performance (AUC, sensitivity, and specificity) and resulting feature dimensionality are reported for a range of univariate significance thresholds (Pv thresholds), evaluated separately for PD-L1 status and histological subtype classification using the same cross-validation scheme as in the main analysis. The table illustrates the trade-off between feature dimensionality and predictive performance, showing that intermediate thresholds provide the most favorable balance, whereas more permissive thresholds lead to a rapid increase in the number of candidate features without proportional performance gains. Metrics could not be computed for histological subtype prediction at p = 0.1 due to excessive feature dimensionality.

	Pv threshold	# features	AUC	Sensitivity	Specificity
PD-L1	0.005	877	0.66	0.814	0.518
	0.01	885	0.64	0.814	0.518
	**0.05**	**2340**	**0.84**	**0.881**	**0.696**
	0.1	7014	0.82	0.729	0.804
Histological type	0.005	1423	0.7	0.766	0.627
	**0.01**	**2635**	**0.76**	**0.875**	**0.627**
	0.05	21350	0.79	0.812	0.686
	0.1	34806	not computed	not computed	not computed

Supplementary Table 4: Evaluation of various tested classifiers used to predict the PD-L1 status:

Model	AUC	Sensitivity	Specificity
glm	0.5921000	0.5256667	0.6061667
lda	0.6234333	0.5416667	0.5878333
knn	0.7402750	0.5803333	0.7348333
svm	0.7240667	0.6725000	0.7183333
rf	0.8307167	0.6985000	0.8520000
pam	0.6756500	0.5838333	0.6841667
nnet	0.7206222	0.6148333	0.7038333
treebag	0.8174306	0.6838333	0.8128333
rpart	0.7399639	0.6785000	0.6891667

Supplementary Table 5: Evaluation of various tested classifiers used to predict the histological type:

Model	AUC	Sensitivity	Specificity
glm	0.5464286	0.4	0.6666667
lda	0.5333333	0.4	0.6666667
knn	0.6833333	0.6	0.8333333
svm	0.7277778	0.6	0.8333333
rf	0.7666667	0.6	0.8333333
pam	0.7000000	0.5	0.8333333
nnet	0.6619048	0.6	0.7142857
treebag	0.7333333	0.6	0.8333333
rpart	0.6166667	0.4	0.7738095

Supplementary Table 6: Basic clinical features of patients from external validation dataset (n=82).

**Factor (feature)**	**PD-L1 negative (n=21)**	**PD-L1 positive (n=61)**	**p-value***
**Age [years] (median and IQR)**	70 (66 – 76)	71 (66 – 76)	0.7781
**Sex**			
male	17	31	0.03072
female	4	30	
**Histological type**			
adenocarcinoma	10	22	0.4985
squamous	11	39	
**T stage**			
1	2	3	
2	3	10	0.2006
3	4	26	
4	12	22	
**N stage**			
0	5	12	
1	5	5	0.3191
2	6	22	
3	5	22	
**M stage**			
0	9	46	0.1356
1	12	15	
**Clinical stage**			
I	2	3	
II	1	5	0.02756
III	6	38	
IV	12	15	

* p-value from either Mann-Whitney test for numeric values (age) and Chi-square test for categorical data

Supplementary Table 7: Confusion matrices for the performance of the final model against the external validation cohort:

**PD-L1 status**			
		Reference	
		negative	*positive*
Prediction	*negative*	13	12
	positive	8	49
**Histological type**			
		Reference	
		adenocarcinoma	*squamous*
Prediction	*adenocarcinoma*	13	16
	squamous	8	45
